# Mechanisms and applications of camelid variable heavy-chain nanobodies against bacterial and parasitic protozoal pathogens

**DOI:** 10.3389/fimmu.2026.1943866

**Published:** 2026-09-15

**Authors:** Hattan S. Gattan, Thamir A. Alandijany

**Affiliations:** 1 Special Infectious Agents Unit, King Fahd Medical Research Center, King Abdulaziz University, Jeddah, Saudi Arabia; 2 Department of Medical Laboratory Sciences, Faculty of Applied Medical Sciences, King Abdulaziz University, Jeddah, Saudi Arabia

**Keywords:** bacterial infections, camelid nanobodies, immunotherapy, One Health, parasitic infections, single-domain antibodies, VHH

## Abstract

Camelid variable heavy-chain (VHH) nanobodies have emerged as a promising class of single-domain antibodies with significant potential for combating bacterial and parasitic infections. Their small molecular size, high solubility, exceptional physicochemical stability, and ability to recognize cryptic or conformational epitopes inaccessible to conventional antibodies enable unique therapeutic applications. This review summarizes recent advances in the biology, mechanisms of action, engineering, and translational potential of camelid VHH nanobodies against bacterial and parasitic pathogens. In bacterial infections, nanobodies primarily function as anti-virulence agents by neutralizing toxins, inhibiting bacterial adhesion and invasion, disrupting secretion systems, preventing biofilm formation, and targeting antimicrobial resistance determinants such as β-lactamases. In protozoal diseases, they exhibit diverse mechanisms including direct killing, inhibition of host-cell invasion, disruption of surface protein trafficking, interference with metabolic pathways, and blockade of transmission stages. We further discuss current approaches for nanobody generation, recombinant production, molecular engineering, and optimization, together with challenges related to pharmacokinetics, immunogenicity, manufacturing, and clinical translation. Finally, emerging applications involving artificial intelligence-assisted nanobody design, nanotechnology-based delivery systems, One Health strategies, and outbreak preparedness are highlighted. Collectively, camelid VHH nanobodies represent a versatile and highly engineerable platform with considerable potential to address antimicrobial resistance, and accelerate the development of next-generation antimicrobial therapeutics.

## 1 Introduction to biology and structural features of camelid VHH nanobodies

The continuous emergence and re-emergence of bacterial and parasitic infections, together with the growing threat of antimicrobial resistance and complex host–pathogen interactions, necessitate the development of innovative immunotherapeutic strategies. Camelid VHH nanobodies represent the antigen-binding domains of a unique class of antibodies naturally produced by members of the Camelidae family, including camels, llamas, and alpacas (1, 2). Unlike conventional antibodies, these molecules function as independent, single-domain units capable of recognizing antigens with high specificity and affinity (1, 2). Their minimalistic architecture, combined with distinctive structural adaptations, underpins a range of functional advantages that are particularly relevant in the context of infectious diseases (3–5). Understanding the biological origin and structural features of VHH nanobodies is therefore essential for appreciating their expanding role as therapeutic tools.

The VHH domain (~12–15 kDa) retains the canonical immunoglobulin fold consisting of a β-sheet framework stabilized by conserved disulfide bonds (1, 6) (**Figure 1**). However, several distinctive structural features differentiate VHH nanobodies from conventional antibody variable domains, contributing to their unique binding properties and biochemical behavior. One of the most defining characteristics of camelid VHH nanobodies is the presence of an extended complementarity-determining region 3 (CDR3) (7, 8). This elongated loop can protrude outward to access recessed, hidden, or conformational epitopes that are typically inaccessible to conventional antibodies due to steric hindrance (7, 8). This unique binding capability allows nanobodies to target conserved and functionally critical microbial structures, including bacterial toxins, resistance-associated enzymes, adhesins, and parasitic surface antigens. Such properties are particularly advantageous for combating structurally complex pathogens and antimicrobial-resistant organisms (7, 8).

**Figure 1:**
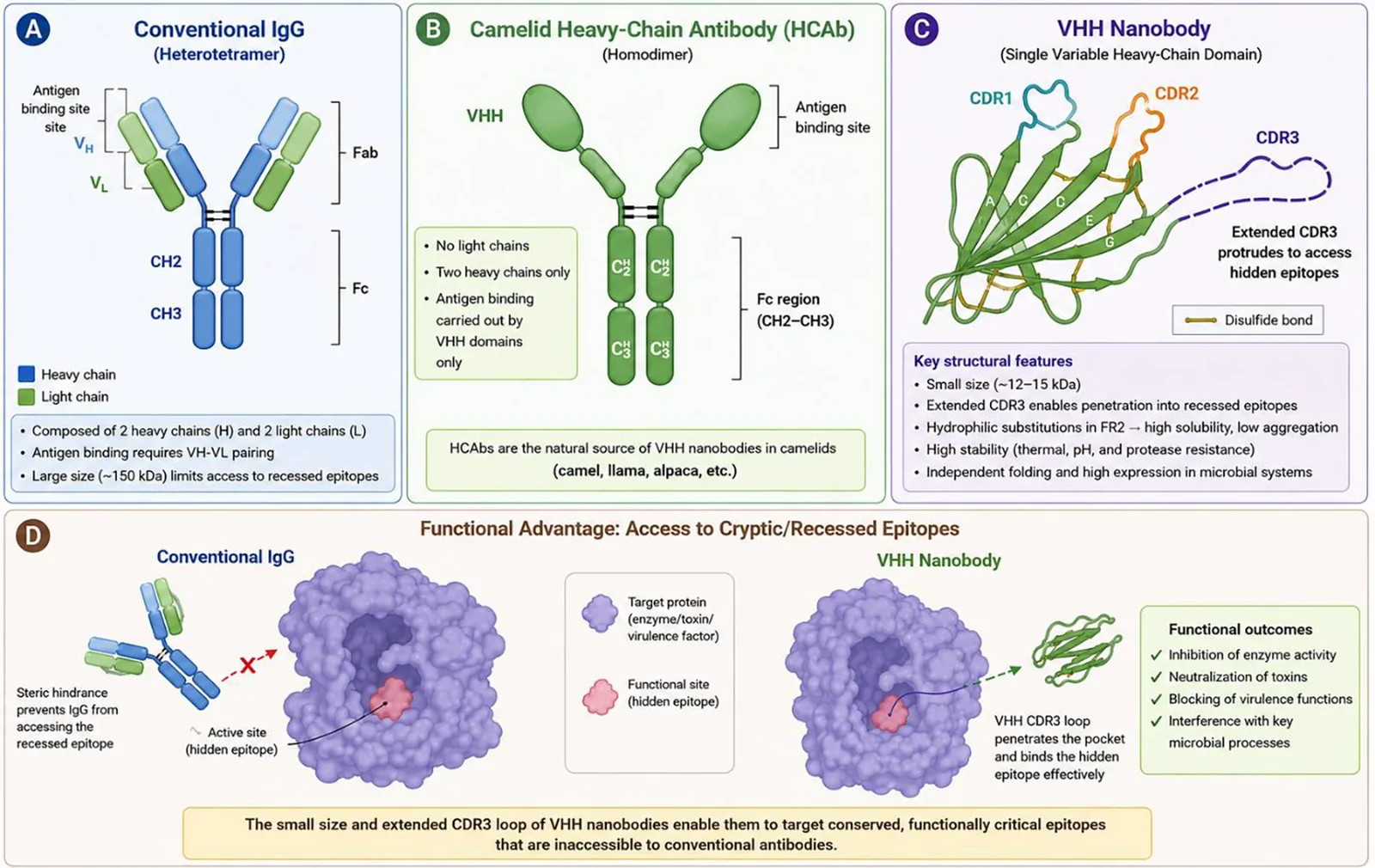
Structural Architecture of Camelid VHH Nanobodies versus Conventional IgG. Comparison between conventional immunoglobulin G (IgG), camelid heavy-chain antibodies (HCAbs), and camelid-derived variable heavy-chain domains (VHH nanobodies). **(A)** Conventional IgG antibodies consist of two heavy chains and two light chains, with antigen recognition mediated through VH–VL pairing within the Fab region. The large molecular size and structural complexity of IgG may restrict access to recessed or cryptic epitopes. **(B)** Camelid HCAbs naturally lack light chains and are composed solely of heavy chains, with antigen binding mediated by single VHH domains. **(C)** Structural representation of a camelid VHH nanobody highlighting the conserved immunoglobulin fold, elongated complementarity-determining region 3 (CDR3), hydrophilic framework region substitutions, and stabilizing disulfide bonds. The extended CDR3 enables penetration into recessed antigenic sites inaccessible to conventional antibodies. **(D)** Functional comparison illustrating the ability of VHH nanobodies to access cryptic or enzymatic pockets within microbial proteins, enabling inhibition of structurally constrained functional epitopes. Collectively, the small size, high stability, and unique structural adaptations of VHH nanobodies underpin their versatility as antimicrobial agents.

In addition to CDR3 elongation, VHH domains exhibit specific amino acid substitutions within framework region 2 (FR2) that enhance hydrophilicity and reduce the propensity for aggregation (9, 10). These substitutions replace hydrophobic residues, typically involved in VH–VL interactions in conventional antibodies, with more hydrophilic counterparts, thereby stabilizing the VHH domain in the absence of a light chain (9, 10). These framework adaptations contribute to the exceptional solubility and stability of nanobodies. Camelid nanobodies exhibit remarkable physicochemical stability, maintaining functionality under extreme conditions of temperature, pH, and denaturing environments (8, 11–13). Their high solubility, resistance to aggregation, and compatibility with microbial expression systems such as Escherichia coli and yeast facilitate cost-effective large-scale production, making them attractive candidates for therapeutic applications (14–16). Furthermore, their small size enhances tissue penetration and supports alternative delivery approaches, including mucosal and localized administration, which may be beneficial for gastrointestinal, systemic, and intracellular infections (17–19).

Beyond the native monomeric VHH format, nanobodies can be molecularly engineered into multivalent, bispecific, Fc-fused, or intracellular formats to modify their functional properties and expand their therapeutic applications (1, 6, 8, 10, 20, 21). Multivalent and bispecific constructs can increase functional avidity or enable simultaneous recognition of different epitopes or targets, whereas Fc fusion can extend circulatory persistence and introduce Fc-dependent effector functions (1, 6, 8, 10, 20, 21). Other engineering approaches, including affinity maturation, PEGylation, and albumin-binding strategies, can be used to optimize binding, stability, and pharmacokinetic properties (1, 6, 8, 10, 20, 21). These engineered formats should be distinguished from unmodified monomeric VHHs because their biological functions, pharmacokinetics, and manufacturing requirements may differ substantially.

## 2 Antimicrobial activity of camelid VHH nanobodies

Camelid VHH nanobodies have emerged as a versatile antimicrobial platform with demonstrated activity across bacterial, and parasitic pathogens (22–27). Across pathogen classes, nanobodies exert their antimicrobial effects through several core mechanisms. In bacterial systems, nanobodies frequently target virulence factors rather than essential survival pathways, thereby reducing selective pressure for resistance development (22–24). In parasitic infections, their activity extends to both extracellular and intracellular stages, where they can disrupt invasion pathways, metabolic processes, and immune evasion mechanisms (25–27). In addition to their direct antimicrobial effects, nanobodies can modulate host immune responses. Engineered formats, such as Fc-fused constructs, can recruit immune effector mechanisms including antibody-dependent cellular cytotoxicity (ADCC) and complement activation (1, 6). Even in the absence of Fc domains, nanobodies can enhance opsonization or interfere with pathogen-mediated immune suppression, contributing to improved pathogen clearance (1, 6). Collectively, these properties position camelid VHH nanobodies as a highly adaptable and powerful platform for antimicrobial intervention. Their capacity to integrate multiple mechanisms of action, coupled with engineering flexibility and compatibility with diverse delivery systems, underscores their potential as next-generation biologics for combating infectious diseases.

### 2.1 Camelid nanobodies against bacterial infections

Camelid VHH nanobodies have demonstrated considerable potential against a wide range of bacterial pathogens through mechanisms that predominantly target virulence rather than directly inducing bacterial killing (22–24, 28, 29). Their antimicrobial activity includes inhibition of adhesion and invasion, neutralization of toxins, disruption of secretion systems, interference with biofilm formation, and inhibition of resistance-associated enzymes (22–24, 28, 29) (**Figure 2**). Due to their small size and extended CDR3 regions, nanobodies can access structurally restricted epitopes within bacterial proteins that are often inaccessible to conventional antibodies (7, 8). Current applications span both Gram-positive and Gram-negative bacteria, highlighting the versatility of camelid nanobodies as emerging antimicrobial and anti-virulence biologics (**Table 1**).

**Figure 2:**
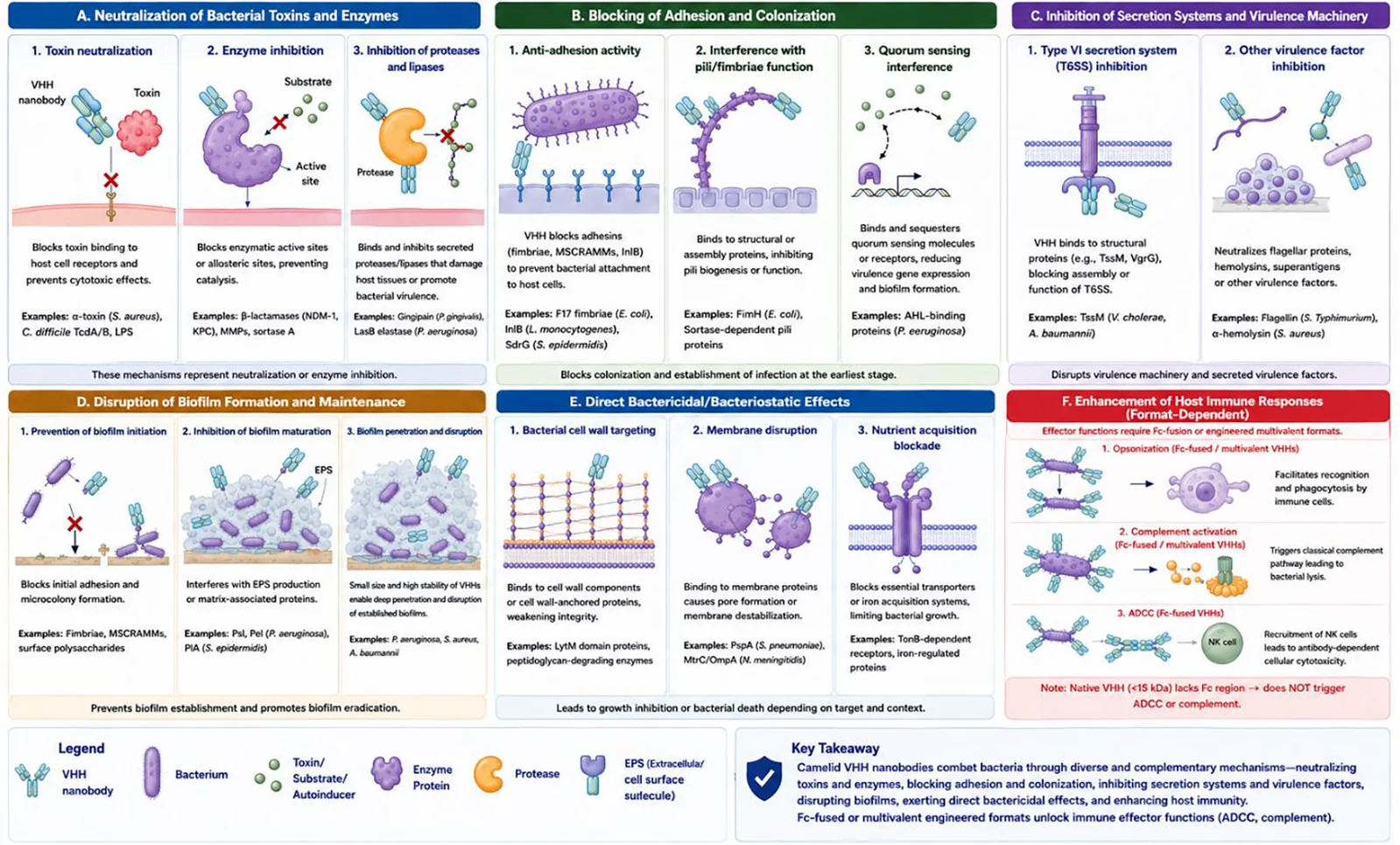
Mechanisms of Anti-bacterial Action of Camelid VHH Nanobodies. Overview of the principal mechanisms by which camelid VHH nanobodies exert antibacterial activity against Gram-positive and Gram-negative bacterial pathogens. Major mechanisms include: **(A)** neutralization of bacterial toxins and inhibition of enzymatic virulence factors, including β-lactamases, proteases, and secreted toxins; **(B)** blockade of bacterial adhesion and colonization through targeting of adhesins, pili, fimbriae, and quorum sensing systems; **(C)** inhibition of secretion systems and virulence machinery, particularly Type VI secretion systems (T6SS) and associated structural proteins; **(D)** disruption of biofilm formation, maturation, and maintenance through interference with extracellular polymeric substances (EPS) and biofilm-associated proteins; **(E)** direct bactericidal or bacteriostatic effects mediated through targeting of cell wall components, membrane proteins, and nutrient acquisition pathways; and **(F)** enhancement of host immune responses through opsonization, complement activation, and antibody-dependent cellular cytotoxicity (ADCC), particularly in Fc-fused formats. Representative bacterial targets include NDM-1 β-lactamase, F17 fimbriae, Internalin B (InlB), TssM, lipopolysaccharide (LPS), and biofilm-associated proteins. Collectively, these mechanisms highlight the multifunctional nature of camelid nanobodies as anti-virulence and antimicrobial agents with potential applications against multidrug-resistant bacterial pathogens.

**Table 1:** Representative studies of camelid VHH nanobodies against bacterial pathogens.

Study	Gram type	Pathogen	Target antigen	Nanobody format	Experimental model	Main outcome	Study limitation
King et al., 2018 (31)	Gram-positive	Listeria monocytogenes	Internalin B (InlB), c-Met binding cavity	Monomeric VHH (R303, R330, R326)	*In vitro*: purified InlB binding, HeLa invasion (gentamicin protection + microscopy), X-ray complex structures	VHH bind the c-Met site on InlB and competitively inhibit invasion (R303 ~94%, R330 ~75%)	No *in vivo* efficacy; invasion blockade only
Valenciano-Bellido et al., 2023 (24)	Gram-positive	Staphylococcus aureus (incl. MRSA)	Hemoglobin receptors IsdH/IsdB (heme-binding pockets)	Monomeric VHH (VHH6)	*In vitro*: SPR/ITC binding, crystallography, MD simulation, bacterial growth assay	A single VHH blocks Isd-mediated heme acquisition and inhibits S. aureus growth	No animal or clinical data
Lu et al., 2012 (59)	Gram-positive	Erysipelothrix rhusiopathiae	Surface protective antigen A (SpaA-N)	Monomeric VHH (two recombinant clones)	*In vitro*: recombinant SpaA-N; Western blot, ELISA, thermal refolding	Recombinant anti-SpaA VHHs bind SpaA-N with thermostable refolding	Binding/reagent characterization only; no neutralization or efficacy
Wang et al., 2025 (30)	Gram-positive	Streptococcus agalactiae (GBS)	Surface immunogenic protein (Sip)	Monomeric VHH (Nb30)	*In vitro* ELISA binding + *in vivo* GBS-infected tilapia	Nb30 raises tilapia survival (53% to 86%) and lowers organ burden dose-dependently	Efficacy in a fish model; mammalian relevance untested
Nguyen et al., 2015 (22)	Gram-negative	Enteroaggregative Escherichia coli (T6SS)	TssM periplasmic domain (Type VI secretion system)	Monomeric llama VHH (nb25, nb42), periplasmic	*In vitro*: SPR/BLI, size-exclusion, X-ray structure of TssM-Nb complex	Nanobodies disrupt the TssM-TssJ interaction required for T6SS assembly	Requires intracellular (periplasmic) access; no whole-organism efficacy
Payandeh et al., 2014 (29)	Gram-negative	Acinetobacter baumannii	Biofilm-associated protein (Bap), conserved region	Monomeric VHH	*In vitro*: recombinant Bap; phage-ELISA/soluble expression	Recombinant VHH reacts with conserved Bap (immunoreactivity)	Binding only; no antibiofilm or efficacy data
Huen et al., 2019 (60)	Gram-negative	Salmonella enterica	FliC flagellin	Monomeric VHH (Abi-Se07), periplasmic	*In vitro*: BLI binding, soft-agar motility, HeLa invasion; ex vivo tissue	Anti-FliC VHH impairs motility and reduces host-cell invasion	No animal or clinical study
Hoseinpoor et al., 2014 (61)	Gram-negative	Helicobacter pylori	UreC subunit of urease	Monomeric VHH mutant (affinity-matured HMR23)	*In vitro*: antigen binding + urease-activity inhibition assay	Affinity-matured VHH binds UreC and inhibits urease activity	*In vitro* only; no cellular or *in vivo* evidence
Ochoa & Schmidt, 2023 (20)	Gram-negative	Shigella/Salmonella	IpaD/SipD, IcsA, IcsP virulence factors	Synthetic-library VHH; some as Nb-Fc fusions	*In vitro*: yeast-display selection, ELISA, flow cytometry, invasion assay	Cross-reactive synthetic nanobodies bind conserved virulence proteins (preliminary)	Limited data (conference abstract)
Ruano-Gallego et al., 2019 (28)	Gram-negative	Enterohemorrhagic E. coli O157:H7	Translocated intimin receptor (TirM)	Monomeric VHH (TD4); also displayed as Nb-HlyA fusion	*In vitro* ELISA/SPR, HeLa infection; ex vivo human colonic mucosa	VHH blocks intimin-Tir attachment and reduces EHEC adherence to human mucosa	No *in vivo* or clinical data
Dhehibi et al., 2024 (33)	Gram-negative	Escherichia coli (F17+ enterotoxigenic)	F17 fimbriae (F17A shaft, F17G adhesin)	Bispecific/bivalent VHH (anti-F17G x anti-F17A)	*In vitro*: recombinant antigens; binding + stability characterization	A bispecific anti-F17 nanobody binds both fimbrial subunits (proposed anti-adhesion tool)	No adhesion-inhibition or efficacy assay reported
Ebrahimizadeh et al., 2013 (32)	Gram-negative	Vibrio cholerae O1	Lipopolysaccharide (LPS), Inaba/Ogawa	Monomeric VHH (four clones)	*In vitro* binding + *in vivo* bacterial challenge	Anti-LPS VHH recognizes both serotypes and shows protection in a challenge model	Mechanism of *in vivo* protection not fully defined
Abbady et al., 2011 (34)	Gram-negative	Brucella melitensis	Brucella lysate antigens (not individually defined)	Phage-displayed VHH library (NbBruc clones)	Camel immunization + phage-display panning; *in vitro* binding	A Brucella-immune VHH library yields specific binders	No defined molecular target; no functional/therapeutic data
Conrath et al., 2001 (62)	Gram-negative	Escherichia coli (beta-lactamase model)	beta-lactamases TEM-1 (class A) and BcII (class B)	Monomeric VHH fragments	*In vitro*: purified-enzyme nitrocefin assays + engineered surface-display E. coli	VHHs specifically inhibit TEM-1 and BcII beta-lactamases	Whole-cell effect shown only via engineered surface expression; native periplasmic access not demonstrated
Ben Abderrazek et al., 2024 (23)	Gram-negative	K. pneumoniae, E. coli, E. cloacae (NDM-1+)	NDM-1 metallo-beta-lactamase	Monomeric VHH (Nb-NDM-1 clones)	*In vitro*: recombinant NDM-1 binding + enzyme kinetics	Uncompetitive NDM-1 inhibitors, IC50 ~8.5-25 nM	Purified enzyme only; no whole-cell re-sensitization (NDM-1 is periplasmic/anchored)
Jimenez et al., 2015 (35)	Gram-negative	E. coli/Pseudomonas putida (model bacteria)	NicX (2,5-dihydroxypyridine dioxygenase) active site	Intracellular thioredoxin-fused VHH (intrabody)	In-cell conditional intrabody expression in bacteria	Active-site intrabody produces a phenotypic metabolic knockout *in vivo*	Requires intracellular expression; proof-of-concept in model bacteria

#### 2.1.1 Activity against gram-positive bacteria

In Gram-positive systems, camelid nanobodies have demonstrated clear *in vivo* efficacy, particularly through disruption of host–pathogen interactions and invasion pathways. In *Streptococcus agalactiae*, the nanobody Nb30 provides a notable example of effective *in vivo* protection in tilapia (30). Treatment resulted in a significant increase in survival rates, rising from 53% in untreated controls to 86% in treated groups, accompanied by substantial reductions in bacterial burden across multiple organs, including spleen, liver, and brain (30). These reductions were dose-dependent, indicating a direct relationship between nanobody concentration and antimicrobial effect. Mechanistically, this activity is mediated through competitive inhibition of the surface immunogenic protein (Sip), thereby blocking interactions with host receptors and limiting bacterial colonization and systemic dissemination (30).

Similarly, in *Listeria monocytogenes*, nanobodies targeting Internalin B (InlB) effectively inhibit bacterial invasion *in vitro* (31). Structural analyses have demonstrated that these nanobodies bind to the cMet interaction site on InlB, thereby preventing receptor-mediated endocytosis (31). This targeted disruption of invasion machinery represents a classical anti-virulence strategy, whereby infection establishment is impaired without directly affecting bacterial viability (31). Collectively, these findings highlight the capacity of nanobodies to interfere with critical virulence determinants in Gram-positive bacteria, resulting in reduced infectivity and improved host outcomes.

#### 2.1.2 Activity against gram-negative bacteria

In Gram-negative bacteria, nanobody applications have primarily focused on targeting enzymatic resistance mechanisms, secretion systems, and surface structures involved in virulence and host interaction. A prominent example is the inhibition of New Delhi metallo-β-lactamase-1 (NDM-1), a key enzyme responsible for carbapenem resistance (23). Nanobodies such as Nb17NDM-1 exhibit potent inhibitory activity at nanomolar concentrations, functioning through uncompetitive inhibition mechanisms that disrupt enzyme activity (23). This inhibition has important therapeutic implications, as it may restore susceptibility to β-lactam antibiotics in resistant strains. Whole-cell and *in vivo* re-sensitization remain to be demonstrated (23).

Nanobodies have also been shown to interfere with bacterial secretion systems. In enteroaggregative *Escherichia coli*, high-affinity nanobodies targeting the TssM component of the Type VI secretion system (T6SS) disrupt protein–protein interactions required for system assembly (22). This results in impaired toxin secretion and reduced competitive fitness, underscoring the ability of nanobodies to target complex virulence machinery.

In addition, nanobody-mediated neutralization of surface structures has been reported. Anti-lipopolysaccharide (LPS) nanobodies targeting *Vibrio cholerae* demonstrated *in vivo* protective effects, likely through neutralization of endotoxin activity and interference with host–pathogen interactions (32). Similarly, bispecific nanobodies targeting fimbrial subunits (F17A and F17G) in *Escherichia coli* inhibit bacterial adhesion, thereby reducing colonization potential (33). Nanobodies against biofilm-associated proteins in *Acinetobacter baumannii* and antigen-specific binders against *Brucella melitensis* further illustrate the breadth of potential targets, although quantitative efficacy data remain limited for several of these systems (29, 34).

Furthermore, nanobodies targeting bacterial metabolic enzymes, such as 2,5-dihydroxypyridine dioxygenase (NicX), have demonstrated the ability to disrupt intracellular metabolic pathways, leading to reduced bacterial fitness (35). These findings expand the scope of nanobody applications beyond surface and secreted targets to include intracellular enzymatic processes.

#### 2.1.3 Mechanistic perspectives, AMR implications, and current limitations

Across both Gram-positive and Gram-negative systems, camelid VHH nanobodies primarily function through anti-virulence mechanisms that impair bacterial pathogenicity without necessarily inducing direct bacterial killing (22, 23, 29, 30, 32, 33, 36). These activities include inhibition of adhesins and invasion-associated proteins, neutralization of toxins, disruption of secretion systems, interference with biofilm-associated structures, and suppression of resistance-associated enzymes such as β-lactamases (22, 23, 29, 30, 32, 33, 36). The small molecular size and elongated CDR3 regions of nanobodies enable access to structurally restricted or cryptic epitopes within bacterial proteins, allowing selective targeting of functionally critical virulence determinants.

This mode of action may offer important advantages in the context of antimicrobial resistance (AMR). By targeting bacterial virulence and resistance pathways rather than essential growth processes, nanobodies may impose lower selective pressure for resistance development compared with conventional antibiotics (22, 23, 29, 30, 32, 33, 36). In addition, nanobodies may serve as adjunctive antimicrobial agents capable of restoring antibiotic susceptibility and enhancing therapeutic efficacy through inhibition of resistance enzymes or disruption of host–pathogen interactions. Their engineering flexibility further supports the development of multivalent, bispecific, or Fc-fused constructs with enhanced antimicrobial functionality and immune-mediated activity (15, 20, 37, 38).

Despite these promising findings, several limitations remain. The current evidence base is heterogeneous and largely dominated by *in vitro* investigations, particularly for Gram-negative pathogens, where many studies focus primarily on binding affinity, enzymatic inhibition, or interference with virulence machinery (22, 23, 29, 30, 32, 33, 36). In contrast, robust *in vivo* validation is currently available for only a limited number of bacterial systems, especially Streptococcus agalactiae (30). Consequently, direct comparisons of efficacy across bacterial classes remain challenging, and the translational relevance of several experimental findings has yet to be fully established. Nevertheless, the broad mechanistic diversity, high specificity, and engineering adaptability of camelid VHH nanobodies underscore their growing potential as next-generation antimicrobial and anti-virulence biologics. Their ability to target conserved bacterial determinants and resistance-associated pathways highlights their promise for addressing multidrug-resistant bacterial infections and the escalating global burden of AMR.

### 2.2 Camelid nanobodies against parasitic infections

Parasitic infections, caused by a diverse range of protozoa and helminths, remain a major global health burden, particularly in low- and middle-income regions (39). The complexity of parasite life cycles, antigenic variation, and intracellular survival strategies pose significant challenges for conventional therapeutics and vaccine development (39). In this context, camelid VHH nanobodies offer a promising alternative due to their small size, high specificity, and ability to access structurally constrained or hidden epitopes (25–27, 40, 41). Their adaptability also enables targeting of both extracellular and intracellular stages of parasites, expanding their potential applications in antiparasitic therapy (25–27, 40, 41) (**Figure 3** and **Table 2**).

**Figure 3:**
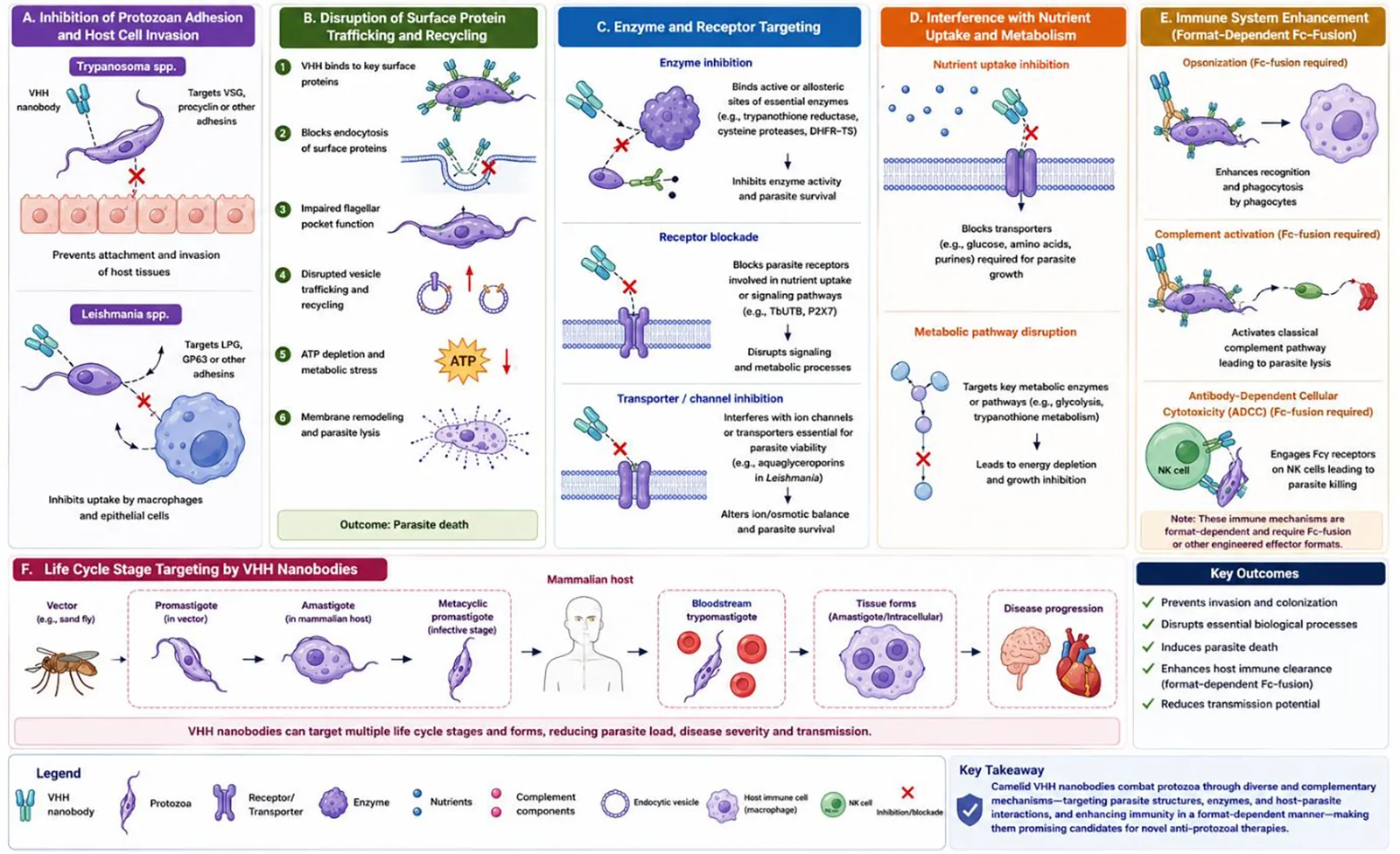
Mechanisms of Anti-Protozoal Action of Camelid VHH Nanobodies. Illustration of the diverse mechanisms by which camelid VHH nanobodies target protozoal pathogens across multiple stages of the life cycle. **(A)** Nanobodies inhibit protozoal adhesion and host-cell invasion by targeting surface molecules involved in attachment and infectivity, including variant surface glycoproteins (VSGs), gp63, and other adhesins. **(B)** In trypanosomes, VHH nanobodies disrupt surface protein trafficking and VSG recycling, leading to blockade of endocytosis, enlargement of the flagellar pocket, impaired vesicular trafficking, ATP depletion, membrane remodeling, and protozoa lysis. **(C)** Nanobodies target essential protozoal enzymes, receptors, ion channels, and transporters through direct or allosteric inhibition, thereby disrupting metabolic pathways and cellular signaling. **(D)** Interference with nutrient uptake and metabolic homeostasis inhibits protozoa growth and survival by targeting transport systems and glycolytic or mitochondrial processes. **(E)** Nanobodies may additionally enhance host immune responses through opsonization, complement activation, and antibody-dependent cellular cytotoxicity (ADCC). **(F)** The figure further highlights the ability of VHH nanobodies to target multiple protozoal developmental stages, including bloodstream, intracellular, and vector-associated forms, thereby reducing burden and transmission potential. Representative protozoa include Trypanosoma spp., Leishmania spp., and Plasmodium spp. Collectively, these mechanisms demonstrate the broad therapeutic potential of camelid VHH nanobodies in antiprotozoal intervention strategies.

**Table 2:** Representative studies of Camelid VHH nanobodies against parasitic pathogens.

Study	Protozoa	Target antigen	Stage	Nanobody format	Experimental model	Main outcome	Study limitation
Stijlemans et al., 2011 (25)	Trypanosoma brucei (AnTat1.1)	Variant surface glycoprotein (VSG)	Bloodstream form	Monomeric VHH; bivalent and human-IgG1-Fc constructs also tested	*In vitro* live-parasite assays + *in vivo* mouse infection	High-affinity anti-VSG VHHs block endocytosis and are trypanolytic, with therapeutic effect *in vivo*	VSG antigenic variation limits breadth of protection
Caljon et al., 2012 (26)	Trypanosoma brucei (AnTat1.1)	Variant surface glycoprotein (VSG)	Bloodstream form	Monomeric VHH (affinity variants; humanized versions tested)	*In vitro*: SPR affinity measurement + live-parasite killing	Anti-trypanosome activity scales with VHH affinity for VSG	*In vitro* focus; constrained by VSG variation
Stijlemans et al., 2004 (44)	Trypanosoma brucei	Conserved cryptic N-linked carbohydrate epitope of VSG	Bloodstream form	Monomeric VHH (cAb-An33); FITC-labeled and beta-lactamase-fusion versions	*In vitro* binding + cultured-parasite detection and enzyme-fusion killing	VHH targets a conserved cryptic VSG epitope, usable for detection and enzyme-fusion killing	Proof-of-concept; enzyme-fusion killing not tested *in vivo*
Hempelmann et al., 2021 (43)	Trypanosoma brucei	VSG surface coat	Bloodstream form	Anti-VSG VHH (format not specified)	*In vitro*: binding on live cells, electron microscopy, structural analysis	VHH-induced macromolecular crowding drives membrane fission and surface-coat remodeling	No *in vivo* evidence
Pinto Torres et al., 2025 (40)	Trypanosoma congolense/T. brucei	Pyruvate kinase (allosteric epitope)	Bloodstream form	Monomeric sdAb42; also expressed as intracellular intrabody	*In vitro* enzyme kinetics, biophysics, X-ray structure; transgenic-parasite survival	Allosteric stabilization of the inactive T-state inhibits parasite pyruvate kinase and impairs fitness	Requires intracellular expression; not a host-administered therapy
Li et al., 2025 (63)	Trypanosoma brucei	T. brucei enolase (TbrENO)	Bloodstream form/enzyme level	Camelid sdAb (format not specified)	*In vitro*: protein biochemistry, biophysics, structural biology	sdAb inhibits TbrENO through a non-active-site mechanism	No cellular or *in vivo* evidence
Abu Alshamat et al., 2020 (27)	Leishmania tropica	Leishmania surface antigen (gp63 proposed)	Promastigotes/intracellular amastigotes	Monomeric VHH (NbLt clones; format not specified)	*In vitro*: parasite lysates, intact parasites, macrophage infection assay	Nanobody treatment reduces the proportion of infected macrophages and parasite burden	*In vitro* only; gp63 target not definitively confirmed
Smolarek et al., 2010 (42)	Plasmodium vivax	Duffy antigen receptor for chemokines (DARC), Fy6 epitope - host receptor	Merozoite invasion (via host receptor)	Monomeric VHH (CA52)	*In vitro*: DARC binding + P. vivax erythrocyte-invasion assays	Anti-DARC VHH blocks P. vivax erythrocyte invasion by occluding the Duffy receptor	Targets a host receptor; *in vitro* only
Lyons et al., 2026 (41)	Plasmodium falciparum	Pfs48/45 (D3 domain)	Sexual stage (gametocyte/gamete)	Fc-fusion nanobodies (A4-Fc, B2-Fc, C3-Fc); monomeric for epitope binning	*In vitro* binding, gametocyte staining, crystal structure, Anopheles standard membrane-feeding assay	Anti-Pfs48/45 Fc-nanobodies block P. falciparum transmission to mosquitoes	Transmission-blocking (not curative); activity requires the Fc format

#### 2.2.1 Direct protozoal killing and growth inhibition

Camelid nanobodies have demonstrated potent antiparasitic activity, particularly against trypanosomes, where direct cytotoxic effects have been established. Specific nanobodies, such as Nb_An05 and Nb_An46, induced complete lysis of *Trypanosoma brucei in vitro*, achieving 100% elimination, with comparable therapeutic efficacy observed *in vivo* following administration of trypanolytic nanobodies (25, 26). Direct killing occurred in a rapid and sequential manner, characterized initially by loss of motility and immobilization within minutes, followed by cellular lysis over the course of several hours (25, 26). Mechanistically, this process was associated with profound morphological alterations, including marked enlargement of the flagellar pocket and disruption of endocytic trafficking, both of which are essential for parasite viability (25, 26).

In contrast, for other parasitic systems, nanobodies predominantly impair infectivity and replication rather than inducing immediate cytolysis. In *Leishmania tropica*, nanobody treatment resulted in a significant reduction in both the proportion of infected macrophages and the intracellular parasite burden (27). Similarly, in *Plasmodium* species, nanobodies inhibited erythrocyte invasion and interfered with transmission by reducing oocyst development within mosquito vectors (41, 42). These findings indicate that nanobody-mediated effects can extend beyond direct killing to include disruption of key stages in protozoal life cycles.

Notably, enzyme-targeting nanobodies can impair protozoal growth through non-lytic mechanisms. For example, the pyruvate kinase-specific nanobody sdAb42 induced dose-dependent growth defects when expressed intracellularly as an intrabody, demonstrating that targeted metabolic interference can significantly reduce protozoal fitness without necessarily triggering immediate cell death (40).

#### 2.2.2 Metabolic and morphological perturbations

Nanobody-mediated antiparasitic activity is frequently accompanied by profound metabolic and structural disruptions. In trypanosomes, treatment with VHH nanobodies resulted in significant reductions in intracellular ATP levels and loss of mitochondrial membrane potential, ultimately leading to energetic collapse and cell death (25). These metabolic disturbances are closely linked to impaired endocytosis, which restricts nutrient uptake and exacerbates cellular stress (26).

Morphological alterations are a hallmark of nanobody action in trypanosomes (25, 26). Targeting of variant surface glycoprotein (VSG) complexes induces progressive cellular swelling, culminating in a transition to a globular morphology (25, 26, 43). This is accompanied by pronounced expansion of the flagellar pocket and the formation of extracellular nanovesicles and nanotubular structures. These membrane remodeling events occur within hours of exposure and reflect a severe disruption of membrane homeostasis and vesicular trafficking (25, 26, 43).

#### 2.2.3 Behavioral disruption

In addition to structural and metabolic effects, nanobodies can induce rapid alterations in protozoal behavior. Certain VSG-targeting nanobodies have been shown to cause an immediate loss of motility in trypanosomes, a process likely linked to interference with the dynamic mobility of VSG molecules on the surface (25, 43). Given that VSG mobility is mechanistically coupled to protozoal propulsion, its disruption results in rapid immobilization, which precedes subsequent cellular dysfunction (25, 43). Together with impaired endocytosis, these behavioral changes contribute significantly to the overall loss of parasite viability.

#### 2.2.4 Mechanisms of action

The mechanisms underlying nanobody-mediated antiparasitic effects are diverse and highly dependent on the molecular target. In trypanosomes, VSG-targeting nanobodies act by binding to epitopes within the dense surface coat, thereby disrupting the highly active recycling of VSG molecules within the flagellar pocket (25, 26). This recycling process is essential for immune evasion and nutrient acquisition; its inhibition leads to endocytic blockade, metabolic collapse, and ultimately parasite death (25, 26). Crucially, the small size and elongated CDR3 of nanobodies enable them to penetrate the densely packed VSG layer, accessing epitopes that are inaccessible to conventional antibodies (43, 44).

In *Plasmodium* species, nanobodies primarily function by blocking host–protozoal interactions. For instance, nanobody CA52 inhibits *Plasmodium vivax* invasion by binding to the Duffy antigen receptor for chemokines (DARC) on erythrocytes, thereby preventing ligand engagement (42). In *Plasmodium falciparum*, nanobodies targeting gamete surface proteins such as Pfs48/45 interfere with fertilization processes, effectively reducing parasite transmission (41).

Importantly, nanobodies can also exert their effects through allosteric mechanisms. The pyruvate kinase-targeting nanobody sdAb42 stabilizes the enzyme in its inactive T-state, preventing transition to the catalytically active R-state (40). These findings highlight the capacity of nanobodies to modulate protein function through non-canonical binding modes, offering novel avenues for therapeutic intervention.

## 3 Production and engineering of camelid VHH nanobodies

The successful application of camelid VHH nanobodies relies on efficient strategies for their generation, selection, recombinant production, and molecular optimization (1, 6, 8, 10, 20, 21) (**Figure 4**). Nanobodies can be obtained from immune, naïve, or synthetic libraries constructed following camelid immunization or *in vitro* diversification of VHH sequences, particularly within the CDR regions (6, 10). High-affinity binders are subsequently isolated using display technologies such as phage display and yeast display through iterative selection and enrichment processes (1, 10). Selected VHHs are then produced using recombinant expression systems including *Escherichia coli*, yeast, mammalian cells, and plant-based platforms, enabling scalable and cost-effective production (1, 21). To improve therapeutic performance, nanobodies may undergo additional engineering strategies such as affinity maturation, multivalent or bispecific construct design, Fc fusion, PEGylation, and albumin-binding modifications to enhance affinity, stability, and circulatory half-life (6, 10, 21). These integrated approaches support the versatility and adaptability of camelid VHH nanobody platforms in antimicrobial research and translational applications.

**Figure 4:**
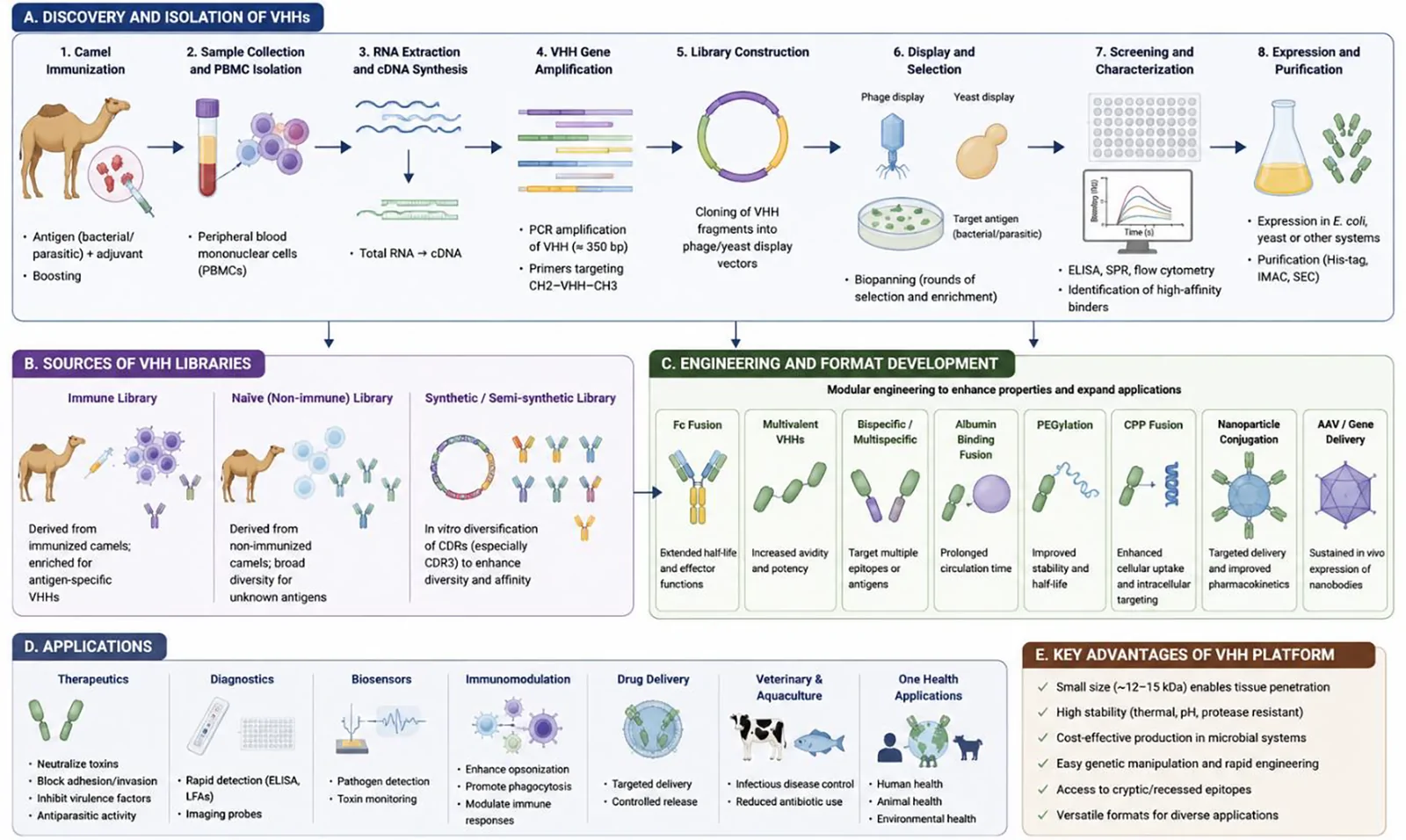
Production and engineering workflow of camelid VHH nanobodies. Schematic overview of the generation, selection, recombinant production, and molecular engineering of camelid VHH nanobodies. **(A)** Nanobody generation begins with camelid immunization using bacterial or parasitic antigens, followed by peripheral blood mononuclear cell (PBMC) isolation, RNA extraction, cDNA synthesis, and amplification of VHH-encoding genes. **(B)** Amplified VHH repertoires are incorporated into immune, naïve, or synthetic/semi-synthetic libraries for downstream screening. **(C)** Engineering strategies including multivalent and bispecific constructs, Fc fusion, PEGylation, albumin-binding approaches, nanoparticle conjugation, and intracellular delivery systems are applied to improve affinity, stability, tissue penetration, and circulatory half-life. **(D)** Engineered nanobodies are subsequently utilized in diverse applications including antibacterial and antiparasitic therapeutics, diagnostics, biosensors, immunomodulation, veterinary medicine, aquaculture, and One Health strategies. Collectively, the figure highlights the versatility, scalability, and engineering flexibility of camelid VHH nanobody platforms for infectious disease applications. **(E)** Key advantages of VHH platform.

### 3.1 Challenges and limitations

Despite their clear advantages, camelid VHH nanobodies face several challenges that may limit their widespread clinical adoption. These include intrinsic pharmacokinetic constraints, potential immunogenicity, manufacturing and regulatory complexities, and barriers in translating promising preclinical findings into clinically approved products. Addressing these limitations is essential for the successful integration of nanobody-based platforms into mainstream infectious disease therapeutics.

#### 3.1.1 Short circulatory half-life

One of the most prominent limitations of VHH nanobodies is their rapid clearance from systemic circulation. Due to their small molecular size (~12–15 kDa), nanobodies are efficiently filtered by the kidneys and eliminated through renal excretion. This results in a relatively short plasma half-life, which may reduce their therapeutic efficacy, particularly in systemic infections requiring sustained exposure (45, 46). While this property can be advantageous for certain applications, such as diagnostic imaging or localized therapies, it presents a challenge for systemic treatment (45–47). To overcome this limitation, various engineering strategies have been developed, including fusion to Fc domains, conjugation with polyethylene glycol (PEGylation), and incorporation of albumin-binding motifs (45, 46). These approaches increase molecular size and prolong circulation time; however, they may also introduce additional complexity in production and regulatory evaluation.

#### 3.1.2 Immunogenicity considerations

Although camelid nanobodies generally exhibit low immunogenicity due to their structural similarity to human antibody variable domains, the risk of immune responses cannot be completely excluded (6, 45, 48). The presence of non-human sequences within the VHH framework may lead to the generation of anti-drug antibodies (ADAs), particularly following repeated administration (6, 49). To mitigate this risk, humanization strategies have been employed, involving the modification of framework regions to more closely resemble human VH sequences while preserving antigen-binding specificity (6, 49). However, such modifications must be carefully optimized to avoid compromising stability or affinity. Additionally, the immunogenic potential may vary depending on the route of administration, dosing regimen, and patient population, necessitating thorough evaluation during clinical development (45, 48).

#### 3.1.3 Manufacturing and regulatory barriers

Although nanobodies can be produced efficiently in microbial expression systems, scaling up production to meet clinical-grade standards introduces several challenges (1, 50). Ensuring consistent product quality, purity, and stability is critical, particularly for complex engineered formats such as multivalent or Fc-fused constructs (51, 52). From a regulatory perspective, nanobody-based therapeutics represent a relatively novel class of biologics, and standardized guidelines for their evaluation are still evolving. Regulatory agencies require comprehensive characterization of pharmacokinetics, immunogenicity, toxicity, and manufacturing processes. The incorporation of non-traditional features, such as multivalency, fusion partners, or alternative delivery systems, may further complicate regulatory approval pathways (48, 51). In addition, the integration of nanobodies into advanced delivery platforms, such as viral vectors or nanoparticle systems, introduces additional layers of regulatory scrutiny related to safety, biodistribution, and long-term effects (48, 53).

#### 3.1.4 Translation from preclinical to clinical use

While numerous studies have demonstrated the efficacy of camelid nanobodies *in vitro* and in animal models, successful translation into clinical applications remains limited (54). Differences in physiology, immune responses, and disease progression between animal models and humans can affect therapeutic outcomes and complicate dose optimization (19, 55–58). Moreover, challenges related to delivery, pharmacokinetics, and large-scale production must be addressed before clinical implementation. Clinical trials are required to establish safety, efficacy, and optimal dosing strategies, which can be time-consuming and resource-intensive.

Another consideration is the need for standardized evaluation frameworks to compare nanobody-based therapies with existing treatments. Without clear benchmarks and clinical endpoints, it may be difficult to demonstrate added value over established therapeutics. Overall, while camelid VHH nanobodies hold significant promise, overcoming these challenges is critical for their successful clinical translation. Continued advancements in protein engineering, manufacturing technologies, and regulatory science will be essential to unlock their full potential in the prevention and treatment of infectious diseases.

Collectively, these limitations extend beyond pharmacokinetics and manufacturing to include target accessibility, antigenic variation and potential escape, format-dependent functionality, and the variable maturity of the available experimental evidence. These challenges, and their differing implications for bacterial and parasitic protozoal applications, are summarized in, **Table 3**.

**Table 3:** Key challenges and limitations of VHH nanobody applications against bacterial and parasitic protozoal pathogens.

Challenge domain	Bacterial pathogens	Parasitic pathogens
Target accessibility and biological compartment	Surface-exposed adhesins or toxins are tractable, but targets in the periplasm, cytosol, or biofilm can be inaccessible to an externally administered VHH. The NDM-1 study, for example, demonstrated inhibition of recombinant enzyme rather than a whole-cell susceptibility phenotype (23).	Target access varies across host and parasite compartments, developmental stages, and—in vector-borne infections—the vertebrate host and vector. For blood-stage trypanosomes, VHHs can reach epitopes within the dense VSG coat, but the coat is dynamically recycled (25).
Antigen diversity, immune evasion, and escape	Single-target anti-virulence strategies can lose activity if the target epitope varies, is masked, or is no longer expressed in the relevant infection state; this is a general vulnerability of highly specific antibody recognition (64).	Antigenic variation is an explicit obstacle for African trypanosomes: a large VSG repertoire changes the antigenic surface and delays antibody-mediated clearance (43).
Format–mechanism alignment	A monomeric VHH may block adhesion, neutralize a toxin, or inhibit an accessible enzyme, but it does not intrinsically recruit Fc-mediated immune effector functions. Such functions require an engineered Fc-containing or cell-engaging format (37).	Format can materially change activity: anti-VSG trypanolysis required low molecular weight, monovalency, and high affinity, whereas malaria Pfs48/45 VHHs were Fc-fused to extend exposure (25, 41).
Pharmacokinetics, exposure, and dosing	Short systemic exposure can limit treatment of disseminated infection, while local delivery may be more practical for some accessible bacterial targets. The *in vivo* Sip study was conducted in tilapia at 100 mg/kg, rather than establishing human systemic dosing (30).	Monomeric nanobodies are rapidly cleared renally; the Pfs48/45 study used human Fc fusion to raise molecular mass above the glomerular-filtration threshold and noted the need to consider further half-life extension (41).
Evidence maturity and translational models	The bacterial evidence spans biochemical inhibition, binding assays, and a limited number of infection models. A strong example is Nb30 in infected tilapia, where bacterial burden fell and survival increased from 53% to 86%; this does not establish efficacy in mammalian infection (30).	Some systems show functional proof of concept—VSG nanobodies lysed trypanosomes *in vitro* and *in vivo*, while Pfs48/45 VHH-Fcs reduced mosquito transmission—but these models address different parasite stages and endpoints (25, 41).
Manufacturing, library provenance, and product consistency	Immune, naïve, and synthetic libraries offer different routes to binders; selection speed does not itself demonstrate antimicrobial function or a manufacturable final format (65).	Parasite-targeting VHHs likewise require format-specific expression and purification. The Pfs48/45 program expressed monomeric VHHs in E. coli and VHH-Fc constructs in mammalian cells, illustrating that format changes can alter production requirements (41).
Immunogenicity, safety, and regulatory development	Repeated systemic administration, engineered fusions, and pathogen-specific toxicology remain to be assessed before therapeutic use; low immunogenicity cannot be assumed from the VHH scaffold alone (66).	The same issue applies to prophylactic or therapeutic parasite programs, particularly when Fc fusion or repeat dosing is needed to compensate for rapid clearance (41, 66).

## 4 Future perspectives and emerging directions

Camelid VHH nanobodies are rapidly evolving from niche antibody fragments into a versatile platform with broad implications for infectious disease control. Ongoing advances in nanotechnology, computational biology, and systems-level health frameworks are expanding their potential beyond conventional therapeutic roles. Future developments are expected to focus on enhancing delivery, accelerating discovery pipelines, and integrating nanobodies into global health strategies aimed at emerging and re-emerging infectious threats.

Realizing these prospective applications will, however, require a substantial expansion of the current *in vivo* evidence base. As highlighted by the studies reviewed above, much of the antibacterial and antiprotozoal literature remains based on biochemical, cellular, or other *in vitro* models, while convincing *in vivo* efficacy has been demonstrated for only a limited number of pathogen–nanobody combinations. Future studies should therefore prioritize physiologically relevant animal models to establish pharmacokinetics, biodistribution, effective dosing, toxicity, immunogenicity, durability of protection, and comparative efficacy against existing therapies. Such validation will be particularly important for engineered, multivalent, Fc-fused, intracellular, and nanocarrier-delivered formats, whose behavior *in vivo* may differ substantially from that of conventional monomeric VHHs. Ultimately, progression from mechanistic proof-of-concept to reproducible *in vivo* efficacy will be essential before these emerging approaches can be considered for clinical or veterinary translation.

### 4.1 Nanobody integration with nanotechnology

The convergence of nanobody engineering with nanotechnology offers powerful opportunities to enhance targeting precision, delivery efficiency, and therapeutic efficacy. Nanobodies can be conjugated to nanoparticles, liposomes, or other nanocarriers to enable targeted delivery of antimicrobial agents, nucleic acids, or imaging probes (19, 67, 68). Their small size and high specificity make them ideal targeting ligands for directing nanomaterials to infected tissues or pathogen-specific antigens.

Such nanobody-functionalized systems can improve drug bioavailability, reduce off-target effects, and enable controlled release of therapeutics. In infectious diseases, this approach is particularly valuable for targeting intracellular pathogens or biofilm-associated bacteria, where conventional treatments often fail to achieve effective concentrations. Additionally, nanobody–nanoparticle conjugates can be used in advanced diagnostic platforms, including highly sensitive biosensors and imaging modalities, further bridging the gap between detection and intervention.

### 4.2 AI-guided design and epitope mapping

Artificial intelligence (AI) and machine learning are transforming the way nanobodies are designed, optimized, and evaluated (10, 50, 51, 65, 69). Computational tools can now predict antigen–antibody interactions, identify conserved epitopes, and guide the rational design of nanobody sequences with enhanced affinity and specificity (10, 50, 51, 65, 69).

AI-driven epitope mapping enables the identification of structurally conserved and functionally critical regions across diverse pathogens, facilitating the development of broadly neutralizing nanobodies (10, 50, 51, 65, 69). In addition, in silico modeling and simulation can accelerate affinity maturation and stability optimization, reducing the need for extensive experimental screening (10, 50, 51, 65, 69).

These approaches are particularly valuable in the context of emerging pathogens, where rapid identification of effective binding sites is essential. By integrating structural biology, bioinformatics, and high-throughput sequencing data, AI-guided platforms can significantly shorten the timeline from antigen discovery to functional nanobody development.

### 4.3 One Health applications

The versatility of camelid nanobodies aligns closely with the principles of the One Health approach, which recognizes the interconnectedness of human, animal, and environmental health. Given their origin in camelids and their applicability across a wide range of pathogens, nanobodies are uniquely positioned to bridge these domains (70, 71).

In veterinary medicine, nanobodies can be used to diagnose and treat infections in livestock and wildlife, thereby reducing zoonotic transmission risks (4, 5, 72). In environmental surveillance, they can be incorporated into biosensors for detecting pathogens in water, food, or air (73, 74). Their stability and scalability make them particularly suitable for deployment in diverse ecological settings (70).

Moreover, nanobody-based platforms can support integrated surveillance systems by enabling rapid detection and response to zoonotic pathogens at the human–animal interface (5). This is especially relevant for regions where close contact between humans and animals facilitates the emergence of novel infectious diseases (5).

### 4.4 Outbreak preparedness and rapid response platforms

The increasing emergence of antimicrobial-resistant bacteria and parasitic outbreaks has highlighted the urgent need for flexible, rapidly deployable antimicrobial countermeasures. Camelid VHH nanobodies represent a promising platform for outbreak preparedness due to their rapid development timelines, scalability, stability, and adaptability to newly emerging microbial targets (70, 71).

Nanobody libraries, including immune, naïve, and synthetic repertoires, can be rapidly screened against emerging bacterial virulence factors, antimicrobial resistance determinants, or parasite surface antigens to identify high-affinity binders within a short timeframe (73). Their compatibility with microbial and plant-based expression systems further enables rapid and cost-effective large-scale production during outbreak situations (75). In addition, the exceptional physicochemical stability of nanobodies facilitates stockpiling, transport, and deployment without stringent cold-chain requirements, which is particularly advantageous in resource-limited or field settings (70, 76).

Innovative delivery platforms, including aerosolized formulations, nanoparticle-based systems, and gene-delivery approaches, may provide rapid localized or sustained antimicrobial protection during outbreaks (70, 77). Combined with AI-guided target identification, genomic surveillance, and One Health monitoring systems, camelid nanobodies could support the development of next-generation rapid response platforms for controlling emerging bacterial and parasitic threats (50, 68, 69). Collectively, these advances highlight the growing potential of camelid VHH nanobodies in outbreak preparedness, antimicrobial resistance mitigation, and integrated infectious disease surveillance across human, animal, and environmental health sectors.

## 5 Conclusion

Camelid VHH nanobodies have emerged as a transformative class of antibody fragments with broad applicability across infectious diseases. Their unique structural and functional properties, including small size, high specificity, exceptional stability, and the ability to access cryptic and conserved epitopes, distinguish them from conventional antibodies and underpin their growing relevance in therapeutic contexts. These features enable effective targeting of diverse microbial pathogens, including bacteria and protozoa, through mechanisms such as neutralization, inhibition of host–pathogen interactions, and suppression of virulence factors.

From a translational perspective, the advantages of VHH nanobodies extend beyond their biological activity. Their compatibility with cost-effective microbial expression systems, amenability to molecular engineering, and flexibility in delivery routes, including inhalation, intranasal, and vector-based expression, facilitate scalable development and deployment. Moreover, the ability to generate multivalent and multispecific constructs enhances their potency and breadth, while engineering strategies addressing pharmacokinetic limitations continue to improve their clinical viability. These attributes position nanobodies as a highly adaptable platform capable of addressing current challenges in antimicrobial resistance, emerging infections, and complex pathogen biology.

Looking forward, camelid nanobodies are poised to play an increasingly important role in clinical and public health applications. In therapeutics, their use as standalone agents or in combination with existing treatments offers new avenues for managing infections that are resistant to conventional approaches. Furthermore, their potential incorporation into pandemic preparedness and rapid diagnostic platforms frameworks supported by advances in artificial intelligence, nanotechnology, and One Health strategies highlights their value as rapid-response tools against future infectious threats.

In summary, camelid VHH nanobodies represent a versatile and forward-looking platform with significant potential to reshape the landscape of infectious disease management. Continued interdisciplinary efforts in engineering, clinical development, and regulatory alignment will be essential to fully realize their promise and translate their capabilities into impactful real-world solutions.
